# 3-Oxo-5-(piperidin-1-yl)-2,3-dihydro-1*H*-pyrazole-4-carbonitrile

**DOI:** 10.1107/S1600536811047714

**Published:** 2011-11-16

**Authors:** Wedad M. Al-Adiwish, W. A. Yaacob, D. Adan, Mohamed Ibrahim Mohamed Tahir, Mohammad B. Kassim

**Affiliations:** a School of Chemical Sciences and Food Technology, Faculty of Science and Technology, Universiti Kebangsaan Malaysia, 43600 Selangor, Malaysia; b Department of Chemistry, Faculty of Science, Universiti Putra Malaysia, 43400 UPM Serdang, Selangor, Malaysia

## Abstract

In the title compound, C_9_H_12_N_4_O, the piperidine ring adopts a chair conformation and makes a dihedral angle of 42.49 (11)° with the approximately planar pyrazole moiety [maximum deviation = 0.038 (2) Å]. In the crystal, N—H⋯O and N—H⋯N hydrogen bonds and a weak C—H⋯O inter­action link the mol­ecules into sheets lying parallel to (110).

## Related literature

For pharmacological background, see: Patel *et al.* (1990 ▶); Morimoto *et al.* (1990 ▶). For related structures see: Zaharan *et al.* (2001 ▶); Elgemeie *et al.* (2007 ▶); Gouda *et al.* (2010 ▶); Shelton *et al.* (2011 ▶). For standard bond lengths, see: Allen *et al.* (1987 ▶).
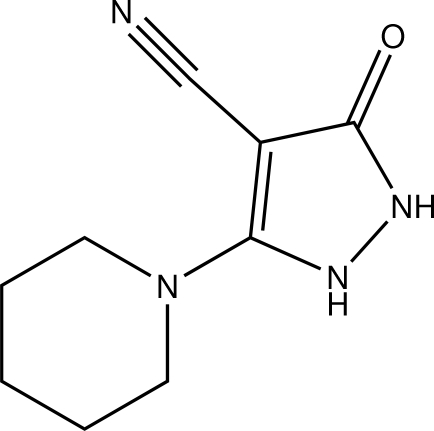


## Experimental

### 

#### Crystal data

C_9_H_12_N_4_O
*M* *_r_* = 192.23
Triclinic, 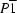

*a* = 7.2667 (5) Å
*b* = 7.9624 (5) Å
*c* = 8.8306 (8) Å
α = 89.280 (6)°
β = 75.934 (7)°
γ = 71.906 (6)°
*V* = 470.01 (6) Å^3^
*Z* = 2
Cu *K*α radiation
μ = 0.77 mm^−1^
*T* = 150 K
0.22 × 0.19 × 0.13 mm

#### Data collection

Oxford Diffraction Gemini diffractometer
Absorption correction: multi-scan (*CrysAlis RED*; Oxford Diffraction, 2006 ▶) *T*_min_ = 0.849, *T*_max_ = 0.906
5083 measured reflections
1803 independent reflections
1627 reflections with *I* > 2σ(*I*)
*R*_int_ = 0.013

#### Refinement

*R*[*F* ^2^ > 2σ(*F* ^2^)] = 0.057
*wR*(*F* ^2^) = 0.166
*S* = 1.11
1803 reflections
127 parameters
H-atom parameters constrained
Δρ_max_ = 0.74 e Å^−3^
Δρ_min_ = −0.61 e Å^−3^

### 

Data collection: *Gemini User Manual* (Oxford Diffraction, 2006 ▶); cell refinement: *CrysAlis RED* (Oxford Diffraction, 2002 ▶); data reduction: *CrysAlis RED*; program(s) used to solve structure: *SHELXS97* (Sheldrick, 2008 ▶); program(s) used to refine structure: *SHELXL97* (Sheldrick, 2008 ▶); molecular graphics: *SHELXTL* (Sheldrick, 2008 ▶); software used to prepare material for publication: *SHELXTL*, *PARST* (Nardelli, 1995 ▶), *PLATON* (Spek, 2009 ▶) and *publCIF* (Westrip, 2010 ▶).

## Supplementary Material

Crystal structure: contains datablock(s) I, global. DOI: 10.1107/S1600536811047714/hb6450sup1.cif

Structure factors: contains datablock(s) I. DOI: 10.1107/S1600536811047714/hb6450Isup2.hkl

Supplementary material file. DOI: 10.1107/S1600536811047714/hb6450Isup3.cml

Additional supplementary materials:  crystallographic information; 3D view; checkCIF report

## Figures and Tables

**Table 1:** Hydrogen-bond geometry (Å, °)

*D*—H⋯*A*	*D*—H	H⋯*A*	*D*⋯*A*	*D*—H⋯*A*
N2—H2⋯N4^i^	0.86	2.32	2.875 (3)	123
N3—H3⋯O1^ii^	0.86	2.07	2.772 (2)	138
C4—H4*A*⋯O1^iii^	0.97	2.54	3.258 (3)	131

Symmetry codes: (i) 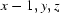; (ii) 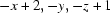; (iii) 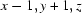.

**Fig. 1.**
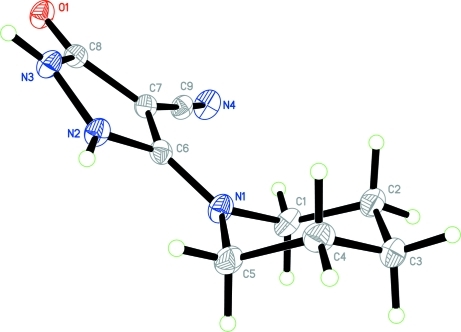
The molecular structure of (I), with displacement ellipsoids are drawn at the 30% probability level.

**Fig. 2.**
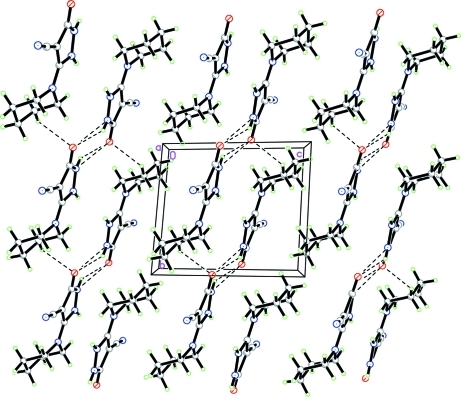
Crystal packing of (I) viewed down the a axis. Hydrogen bonds [N—H···O (x-1, y, z & -x + 2, -y, -z) N—H···N (x-1, y + 1, z)] are drawn as dashed lines.

## supplementary crystallographic information

### Comment

In this paper, we report the synthesis and structure of the new derivative of 3-oxo-5-(piperidin-1-yl)-2,3-dihydro-1*H*- pyrazole-4-carbonitrile. The compound was obtained by cyclization reaction between ethyl 2-cyano-3-(methylthio)-3-(piperidin-1-yl)acrylate and hydrazine.

In the title compound (I), the mean plane of the pyrazole O1/N1/N2/N4/C5/C6/C7/C8/C9 is essentially planar with maximum deviation of -0.038 (2)° for C8 and forms a dihedral angle of 42.49 (11)° with that of the piperidine mean plane N1/C1/C2/C3/C4/C5 (Fig. 1 & Scheme 1). Consequently, a short non-bonding intra D—H..H—*X* contact forms between the N2—H2 of the pyrazole and the H5B—C5 of the piperidine moeities.

The carbonyl C8=O1 [1.246 (2)] and C6=C7 [1.407 (3) Å] are longer than the average [C=O(1.200 Å)] and C=C [(1.340 Å)] bond lengths, respectively. Whereas the C6—N2 [1.363 (2) Å] and C8—N3 [1.375 (2) Å] bond lengths are shorter than the average C—N [(1.47 Å)] indicative of electron-donating effects of the amino groups. Other bond lengths and angle in the molecules are in the normal ranges (Allen *et al.*,1987).

In the crystal, intermolecular hydrogen bonds N2—H2···O4 and N3—H3···O1 and a weak C4—H4···O1 interaction link the molecules forming a two-dimensional polymeric network parallel to (110) (Fig. 2).

### Experimental

A mixture of ethyl 2-cyano-3-(methylthio)-3-(piperidin-1-yl)acrylate (4 mmol) and hydrazine hydrate (4 mmol) was heated on a water-bath for 2 h. Then, ethanol (20 ml) was added and the mixture was refluxed for another 2 h. The solvent was evaporated and the product was collected, washed with ethanol, and dried. Colourless blocks of (I) were formed by slow evaporation of the compound from ethanol solution. Yield = 90%.

### Refinement

H atoms of both C and N atoms were positioned geometrically and allowed to ride on their parent atoms, with *U*_iso_ = 1.2*U*_eq_ (C) for CH_2_ 0.97 Å. Hydrogen atoms attached to N were also positioned geometrically and allowed to ride on their parent atoms and with *U*_iso_(H) = 1.2*U*_eq_(N) for N–H 0.86 Å.

### Crystal data

**Table:**

C_9_H_12_N_4_O	*Z* = 2
*M**_r_* = 192.23	*F*(000) = 204
Triclinic, *P*1	*D*_x_ = 1.358 Mg m^−^^3^
Hall symbol: -P 1	Melting point: 527 K
*a* = 7.2667 (5) Å	Cu *K*α radiation, λ = 1.54178 Å
*b* = 7.9624 (5) Å	Cell parameters from 3129 reflections
*c* = 8.8306 (8) Å	θ = 5–71°
α = 89.280 (6)°	µ = 0.77 mm^−^^1^
β = 75.934 (7)°	*T* = 150 K
γ = 71.906 (6)°	Block, colourless
*V* = 470.01 (6) Å^3^	0.22 × 0.19 × 0.13 mm

### Data collection

**Table:**

Oxford Diffraction Gemini diffractometer	1803 independent reflections
Radiation source: fine-focus sealed tube	1627 reflections with *I* > 2σ(*I*)
graphite	*R*_int_ = 0.013
ω/2θ scans	θ_max_ = 70.9°, θ_min_ = 5.2°
Absorption correction: multi-scan (*CrysAlis RED*; Oxford Diffraction, 2006)	*h* = −8→8
*T*_min_ = 0.849, *T*_max_ = 0.906	*k* = −9→9
5083 measured reflections	*l* = −10→10

### Refinement

**Table:**

Refinement on *F*^2^	Primary atom site location: structure-invariant direct methods
Least-squares matrix: full	Secondary atom site location: difference Fourier map
*R*[*F*^2^ > 2σ(*F*^2^)] = 0.057	Hydrogen site location: inferred from neighbouring sites
*wR*(*F*^2^) = 0.166	H-atom parameters constrained
*S* = 1.11	*w* = 1/[σ^2^(*F*_o_^2^) + (0.0971*P*)^2^ + 0.2641*P*] where *P* = (*F*_o_^2^ + 2*F*_c_^2^)/3
1803 reflections	(Δ/σ)_max_ < 0.001
127 parameters	Δρ_max_ = 0.74 e Å^−^^3^
0 restraints	Δρ_min_ = −0.61 e Å^−^^3^

### Special details

**Table:**

Experimental. The crystal was placed in the cold stream of an Oxford Cryosystems open-flow nitrogen cryostat (Cosier & Glazer, 1986) with a nominal stability of 0.1 K.Cosier, J. & Glazer, A.M., 1986. J. Appl. Cryst. 105 107.
Geometry. All e.s.d.'s (except the e.s.d. in the dihedral angle between two l.s. planes) are estimated using the full covariance matrix. The cell e.s.d.'s are taken into account individually in the estimation of e.s.d.'s in distances, angles and torsion angles; correlations between e.s.d.'s in cell parameters are only used when they are defined by crystal symmetry. An approximate (isotropic) treatment of cell e.s.d.'s is used for estimating e.s.d.'s involving l.s. planes.
Refinement. Refinement of *F*^2^ against ALL reflections. The weighted *R*-factor *wR* and goodness of fit *S* are based on *F*^2^, conventional *R*-factors *R* are based on *F*, with *F* set to zero for negative *F*^2^. The threshold expression of *F*^2^ > σ(*F*^2^) is used only for calculating *R*-factors(gt) *etc*. and is not relevant to the choice of reflections for refinement. *R*-factors based on *F*^2^ are statistically about twice as large as those based on *F*, and *R*- factors based on ALL data will be even larger.

### Fractional atomic coordinates and isotropic or equivalent isotropic displacement parameters (Å^2^)

**Table:**

	*x*	*y*	*z*	*U*_iso_*/*U*_eq_	
O1	1.2334 (2)	0.03312 (18)	0.39572 (17)	0.0334 (4)	
N1	0.8756 (2)	0.6211 (2)	0.2901 (2)	0.0306 (4)	
N2	0.8011 (2)	0.3667 (2)	0.38329 (19)	0.0273 (4)	
H2	0.6730	0.4130	0.4070	0.033*	
N3	0.9078 (2)	0.1884 (2)	0.39566 (19)	0.0285 (4)	
H3	0.8563	0.1043	0.4146	0.034*	
N4	1.4774 (3)	0.3703 (3)	0.2487 (2)	0.0397 (5)	
C1	1.0124 (3)	0.7167 (3)	0.2139 (3)	0.0349 (5)	
H1A	1.1496	0.6418	0.2014	0.042*	
H1B	0.9907	0.8218	0.2789	0.042*	
C2	0.9771 (4)	0.7692 (3)	0.0547 (3)	0.0394 (5)	
H2A	1.0165	0.6635	−0.0144	0.047*	
H2B	1.0596	0.8413	0.0094	0.047*	
C3	0.7585 (4)	0.8722 (3)	0.0673 (3)	0.0424 (6)	
H3A	0.7234	0.9851	0.1253	0.051*	
H3B	0.7386	0.8954	−0.0366	0.051*	
C4	0.6237 (3)	0.7685 (3)	0.1497 (3)	0.0380 (5)	
H4A	0.4847	0.8390	0.1628	0.046*	
H4B	0.6491	0.6610	0.0865	0.046*	
C5	0.6632 (3)	0.7218 (3)	0.3084 (2)	0.0333 (5)	
H5A	0.6282	0.8293	0.3742	0.040*	
H5B	0.5808	0.6517	0.3588	0.040*	
C6	0.9374 (3)	0.4540 (2)	0.3267 (2)	0.0249 (4)	
C7	1.1293 (3)	0.3363 (2)	0.3210 (2)	0.0252 (4)	
C8	1.1054 (3)	0.1702 (2)	0.3729 (2)	0.0262 (4)	
C9	1.3185 (3)	0.3601 (3)	0.2816 (2)	0.0289 (5)	

### Atomic displacement parameters (Å^2^)

**Table:**

	*U*^11^	*U*^22^	*U*^33^	*U*^12^	*U*^13^	*U*^23^
O1	0.0258 (7)	0.0273 (7)	0.0420 (8)	−0.0030 (6)	−0.0067 (6)	0.0098 (6)
N1	0.0276 (9)	0.0285 (9)	0.0356 (9)	−0.0062 (7)	−0.0116 (7)	0.0092 (7)
N2	0.0200 (8)	0.0254 (8)	0.0343 (9)	−0.0032 (6)	−0.0082 (6)	0.0071 (6)
N3	0.0254 (8)	0.0232 (8)	0.0371 (9)	−0.0070 (7)	−0.0092 (7)	0.0072 (6)
N4	0.0283 (10)	0.0412 (11)	0.0545 (11)	−0.0131 (8)	−0.0169 (8)	0.0118 (8)
C1	0.0353 (11)	0.0276 (10)	0.0462 (12)	−0.0126 (9)	−0.0152 (9)	0.0095 (9)
C2	0.0445 (13)	0.0297 (11)	0.0387 (12)	−0.0095 (9)	−0.0038 (9)	0.0084 (9)
C3	0.0532 (14)	0.0321 (11)	0.0331 (11)	−0.0007 (10)	−0.0116 (10)	0.0076 (9)
C4	0.0364 (11)	0.0332 (11)	0.0388 (11)	0.0013 (9)	−0.0154 (9)	0.0018 (9)
C5	0.0295 (11)	0.0274 (10)	0.0382 (11)	−0.0016 (8)	−0.0093 (8)	0.0061 (8)
C6	0.0271 (10)	0.0274 (10)	0.0215 (8)	−0.0078 (8)	−0.0095 (7)	0.0035 (7)
C7	0.0244 (9)	0.0259 (10)	0.0257 (9)	−0.0066 (7)	−0.0090 (7)	0.0029 (7)
C8	0.0255 (9)	0.0261 (9)	0.0252 (9)	−0.0050 (7)	−0.0071 (7)	0.0016 (7)
C9	0.0298 (11)	0.0269 (10)	0.0318 (10)	−0.0070 (8)	−0.0141 (8)	0.0065 (8)

### Geometric parameters (Å, °)

**Table:**

O1—C8	1.246 (2)	C2—H2A	0.9700
N1—C6	1.329 (3)	C2—H2B	0.9700
N1—C1	1.467 (3)	C3—C4	1.520 (3)
N1—C5	1.471 (3)	C3—H3A	0.9700
N2—C6	1.376 (2)	C3—H3B	0.9700
N2—N3	1.408 (2)	C4—C5	1.517 (3)
N2—H2	0.8600	C4—H4A	0.9700
N3—C8	1.362 (3)	C4—H4B	0.9700
N3—H3	0.8600	C5—H5A	0.9700
N4—C9	1.148 (3)	C5—H5B	0.9700
C1—C2	1.520 (3)	C6—C7	1.407 (3)
C1—H1A	0.9700	C7—C9	1.406 (3)
C1—H1B	0.9700	C7—C8	1.442 (3)
C2—C3	1.521 (3)		
			
C6—N1—C1	123.24 (17)	C2—C3—H3B	109.5
C6—N1—C5	122.91 (17)	H3A—C3—H3B	108.1
C1—N1—C5	113.60 (16)	C5—C4—C3	109.84 (19)
C6—N2—N3	108.06 (14)	C5—C4—H4A	109.7
C6—N2—H2	126.0	C3—C4—H4A	109.7
N3—N2—H2	126.0	C5—C4—H4B	109.7
C8—N3—N2	109.28 (15)	C3—C4—H4B	109.7
C8—N3—H3	125.4	H4A—C4—H4B	108.2
N2—N3—H3	125.4	N1—C5—C4	110.09 (17)
N1—C1—C2	109.99 (17)	N1—C5—H5A	109.6
N1—C1—H1A	109.7	C4—C5—H5A	109.6
C2—C1—H1A	109.7	N1—C5—H5B	109.6
N1—C1—H1B	109.7	C4—C5—H5B	109.6
C2—C1—H1B	109.7	H5A—C5—H5B	108.2
H1A—C1—H1B	108.2	N1—C6—N2	120.17 (17)
C1—C2—C3	111.40 (19)	N1—C6—C7	132.01 (18)
C1—C2—H2A	109.3	N2—C6—C7	107.82 (16)
C3—C2—H2A	109.3	C9—C7—C6	131.41 (17)
C1—C2—H2B	109.3	C9—C7—C8	121.20 (17)
C3—C2—H2B	109.3	C6—C7—C8	107.35 (16)
H2A—C2—H2B	108.0	O1—C8—N3	124.15 (18)
C4—C3—C2	110.68 (18)	O1—C8—C7	129.26 (18)
C4—C3—H3A	109.5	N3—C8—C7	106.58 (16)
C2—C3—H3A	109.5	N4—C9—C7	176.5 (2)
C4—C3—H3B	109.5		

### Hydrogen-bond geometry (Å, °)

**Table:**

*D*—H···*A*	*D*—H	H···*A*	*D*···*A*	*D*—H···*A*
N2—H2···N4^i^	0.86	2.32	2.875 (3)	123
N3—H3···O1^ii^	0.86	2.07	2.772 (2)	138
C4—H4A···O1^iii^	0.97	2.54	3.258 (3)	131

Symmetry codes: (i) *x*−1, *y*, *z*; (ii) −*x*+2, −*y*, −*z*+1; (iii) *x*−1, *y*+1, *z*.
